# Methods of cervical ripening in induction of labour: an individual participant data network meta-analysis of randomised controlled trials (CIRCLE-NMA) study protocol

**DOI:** 10.1136/bmjopen-2025-110091

**Published:** 2026-04-13

**Authors:** Nicole Au, Malitha Patabendige, Sol Libesman, Rui Wang, Lyle C Gurrin, Ben W Mol, Wentao Li, the CIRCLE-NMA Collaborative, David M Haas

**Affiliations:** 1 Department of Obstetrics and Gynaecology, Monash Health, Monash University Faculty of Medicine, Nursing and Health Sciences, Clayton, Victoria, Australia; 2 Department of Obstetrics and Gynaecology, St John of God Midland Public and Private Hospitals, Midland, Western Australia, Australia; 3 NextGen Evidence Synthesis Team, Evidence Integration, NHMRC Clinical Trials Centre, The University of Sydney, Sydney, New South Wales, Australia; 4 University of Melbourne School of Population and Global Health, Melbourne, Victoria, Australia; 5 Monash University School of Public Health and Preventive Medicine, Melbourne, Victoria, Australia; 6 National Perinatal Epidemiology and Statistics Unit, Centre for Big Data Research in Health, University of New South Wales Faculty of Medicine, Sydney, New South Wales, Australia

**Keywords:** Network Meta-Analysis, Maternal medicine, Fetal medicine, Randomized Controlled Trial

## Abstract

**Abstract:**

**Introduction:**

Induction of labour (IOL) is a commonly performed obstetric intervention, particularly when delivery is deemed more beneficial than continuing the pregnancy due to maternal or fetal indications. When the cervix is unfavourable for delivery, cervical ripening is performed prior to IOL. A wide variety of mechanical, pharmacological and combination methods are used, but the optimal approach balancing efficacy, safety and patient experience remains uncertain. Conventional aggregate data (AD) meta-analyses lack individual-level data, limiting exploration of patient-level factors for personalised medicine and do not address concerns about the trustworthiness of data presented in peer-reviewed randomised controlled trials (RCTs). This protocol describes an individual participant data (IPD) network meta-analysis (NMA) designed to evaluate and rank cervical ripening methods for IOL using only high quality, trustworthy data.

**Methods and analysis:**

We will identify eligible parallel-group RCTs enrolling pregnant women with a singleton, cephalic fetus at ≥34 weeks’ gestation requiring cervical ripening, through comprehensive searches of Ovid MEDLINE, Embase, Emcare, Scopus, Cochrane Pregnancy and Childbirth Register, WHO International Clinical Trials Registry Platform, clinicaltrials.gov and reference lists of prior reviews. The interventions we consider will be selected via Delphi consensus with international clinical experts. Eligible trial investigators will be invited to contribute de-identified IPD; AD will be used if IPD is unavailable. Trials will be assessed for trustworthiness using the Trustworthiness in RAndomised Clinical Trials checklist and the IPD Integrity Tool, with only eligible studies included in the primary analysis. All statistical analyses will follow a pre-specified statistical analysis plan (SAP) finalised before any analyses are conducted. A two-stage, contrast-based, frequentist IPD-NMA will compare cervical ripening methods for three co-primary outcomes: vaginal birth, composite adverse perinatal outcomes and composite adverse maternal outcomes. Subgroup analyses will assess effect modifiers (eg, parity, age and previous caesarean), with treatment rankings presented using the surface under the cumulative ranking curve and rank-heat plots. Sensitivity analyses will examine the impact of bias, missing data and population criteria.

**Ethics and dissemination:**

This study has been approved by the Monash University Human Research Ethics Committee (No. 48189). IPD will be de-identified and securely transferred for storage on a Monash University-hosted shared network drive. Findings will be disseminated via peer-reviewed publications, conference abstracts and the Cervical Ripening for Induction of Labour Collaborative Evidence Network Meta-Analysis (CIRCLE-NMA) website (https://circlenma.com). Patient and public involvement will guide the communication and interpretation of results.

**PROSPERO registration number:**

CRD420251077464.

**STRENGTHS AND LIMITATIONS OF THIS STUDY**

- The use of individual participant data (IPD) enables standardisation of outcomes and facilitates robust subgroup analyses.
- IPD allows for patient-level adjustment, supporting a more tailored and informed approach to personalised medicine in the future.
- Network meta-analysis (NMA) is an effective way to simultaneously compare and rank multiple interventions across trials.
- The trustworthiness components of this NMA, assessed through Trustworthiness in RAndomised Clinical Trials and the IPD Integrity Tool screening, enhance the reliability of the results.
- The analysis may be limited by the availability of shared IPD. For maternal experience outcomes, which are subjective and currently lack a universal scale, harmonising the data for meaningful analysis may be challenging.

## Introduction and background

 Induction of labour (IOL) is a common obstetric procedure that aims to start labour medically rather than waiting for it to begin naturally. Common reasons for inducing labour include maternal indications, such as hypertensive disorders, diabetes mellitus or the fetus reaching a certain gestational age, as well as fetal indications, such as a diagnosis of fetal growth restriction.^1^,^3^ When the cervix is unripe, cervical ripening is needed before IOL to increase the chance of vaginal birth.^4^ Methods of cervical ripening in IOL can be broadly divided into four categories: mechanical, pharmacological, a combination of both and alternative methods.^5^ Alternative methods such as, for example, nipple stimulation, sexual intercourse, castor oil, acupuncture and homeopathy are not recommended in contemporary obstetric practice.^5 6^

The choice of cervical ripening method is influenced by several factors, such as local clinical guidelines, institutional policies, maternal–fetal well-being, resource implications and the preferences of women. There is a rising interest in using a combination of mechanical and pharmacological methods, such as balloon plus vaginal misoprostol, anticipating the need to balance effectiveness with safety.^7^ Evidence supporting the use of cervical osmotic dilators is re-emerging.^8^ There is, however, still a lack of evidence to determine which method is best, balancing effectiveness, safety, cost and patient experience.

Randomised controlled trials (RCTs) provide high quality evidence for pairwise comparison of IOL methods, and synthesising this evidence is critical for informing clinical practice guidelines. Conventional aggregate data (AD) meta-analyses rely on summary statistics reported in published RCTs. In contrast, individual participant data (IPD) meta-analyses involve the central collection and synthesis of raw data for each participant from eligible trials. This offers several important advantages, including the ability to identify problematic trials, harmonise the definition of both outcomes and covariates across studies, conduct reliable subgroup analyses without the problem of aggregation bias, standardise analytic approaches and apply flexible statistical models. A network IPD meta-analysis further extends these benefits by enabling the simultaneous comparison of multiple treatments within a single analytical framework.^9 10^ Given the significant time and resources required for new trials, IPD network meta-analysis (NMA) provides a practical and robust approach to identify optimal cervical ripening methods for IOL.

Another key motivation for using IPD in evidence synthesis is the growing concern around trial trustworthiness. Recent estimates suggest that up to 30% of published RCT data may be untrustworthy.^11^ These problematic data can meaningfully distort the conclusions of meta-analyses.^12^ A single retracted trial has the potential to contaminate three systematic reviews, each of which may subsequently influence at least three clinical guidelines.^13^ Identifying and excluding problematic trials is, therefore, a critical step in safeguarding the integrity of evidence synthesis, and one that is far more achievable when raw IPD are available for scrutiny.^11^

To address a critical evidence gap, we aim to identify optimal cervical ripening methods in IOL through an IPD-NMA using high quality and trustworthy data. Previous meta-analyses on cervical ripening in IOL have relied on AD for analysis and did not consider the possibility that studies contributing data may have failed trustworthiness checks. Moreover, these studies combined data from women with both favourable and unfavourable cervices, applying a one-size-fits-all approach that overlooks individual variability. This assumption that all methods for cervical ripening perform similarly regardless of maternal characteristics across diverse patient profiles limits their clinical usefulness.^14^ In contrast, an IPD-NMA allows analyses that account for individual-level variability (eg, maternal age and parity), supporting personalised and evidence-based decisions in IOL.

## PICO-based research question

What cervical ripening method(s) for IOL offer the highest effectiveness while ensuring optimal perinatal and maternal safety, and do these preferred method/s differ across subgroups of patients?

Population: pregnant women with a singleton live pregnancy and a cephalic presentation at or after 34 weeks of gestation, regardless of previous caesarean delivery or pre-labour rupture of membranes in the enrolled trial pregnancy, who require cervical ripening in labour induction.

Intervention and relevant comparators: clinically available and emerging cervical ripening methods. An approach based on the Delphi process will be used to determine relevant cervical ripening methods in this IPD-NMA.

Outcomes: three co-primary outcomes will be the proportion of vaginal delivery, a composite measure of adverse perinatal outcomes, and a composite measure of adverse maternal outcomes.

## Objectives

The aims of this study are:

1. To evaluate, compare and rank the effectiveness, perinatal safety, maternal safety and maternal satisfaction for a series of cervical ripening methods for IOL.
2. To identify maternal patient-level factors that modify the effectiveness and neonatal/maternal safety outcomes of different cervical ripening methods in IOL.
3. To assess the trustworthiness of RCTs on cervical ripening methods for IOL and explore how excluding data from studies that fail trustworthiness checks affects the results of evidence synthesis.

## Methods and analysis

This project will be conducted following the methodologies recommended by the Cochrane IPD, Multiple Interventions and Prospective Meta-Analysis Methods Groups.^15^,^17^ The reporting of this protocol is aligned with the NMA protocol guidelines by Wang *et al* and the Preferred Reporting Items for Systematic Reviews and Meta-Analyses (PRISMA) extension for protocols.^18^,^20^ This IPD-NMA has been prospectively registered in PROSPERO (CRD420251077464).

### Eligibility criteria

#### Types of studies

Only individual, parallel-group, RCTs will be included. Cross-over trials, cluster-randomised and quasi-randomised trials will be excluded. Studies available only in abstract form or unpublished will be considered for inclusion if adequate information regarding study design, protocols, participant demographics, outcomes and interventions is provided by the trial authors or available based on study protocols and/or trial registration records. There will be no language restrictions, and both published and unpublished data will be eligible.

#### Type of participants

All women:

- Pregnant with a singleton, cephalic, live fetus of at least 34 weeks gestational age at randomisation.
- Presenting with an unfavourable cervix at randomisation, defined by a modified Bishop score < 6^4^ or an alternative validated measure if well documented.

Data from studies that focused on specific populations of women defined by parity, previous caesarean delivery, setting of treatment (ie, inpatient or outpatient), presence of gestational diabetes mellitus, pre-eclampsia or pre-labour rupture of membranes will also be included.

#### Types of interventions and comparators

The selection of interventions to be included in this NMA will be determined through a Delphi consensus process.^21^ A clinical advisory panel consisting of seven experts in obstetrics and gynaecology from five continents will be invited to participate in this process.^22^ In the first round, the research team will review cervical ripening methods recommended by major international guidelines such as the American College of Obstetricians and Gynecologists, the International Federation of Gynecology and Obstetrics, the National Institute for Health and Care Excellence and the WHO,^423^,^25^ and will supplement this with emerging methods that feature in published research and suggestions from local clinical experts. The compiled list of candidate methods (including dose variations of pharmacological methods) will then be presented to the clinical advisory panel for feedback on the relevance, appropriateness and completeness of the included interventions. Panel members may also suggest additional methods not initially included based on their expertise, practice and geographical locality.

In the second round, based on the findings of the first round, an online survey will be developed and distributed to members of the clinical advisory panel, primarily composed of trialists with experience in RCTs on IOL methods. Panellists will be asked to assess and select interventions that they think should be considered in the IPD-NMA, considering factors such as clinical effectiveness, feasibility of implementation, frequency of use and alignment with current practice. Interventions that receive at least 50% of the vote for inclusion in the IPD-NMA during the second round will advance to the third round.

In the third round of the Delphi process, panel members will receive a summary of the results from the previous rounds. They will be asked to review the results and indicate whether they agree with the selected interventions and outcomes to be included in the final IPD-NMA.

If any concerns are raised or there is notable disagreement among panellists, an additional round may be conducted to reach a consensus. Panel members will be invited to participate in an online clinical advisory group meeting and asked to share their reasons for disagreement. This will allow panel members to reconsider their views based on feedback from others and further discussion.

The potential cervical ripening interventions, including individual and combination methods, to be considered in the first round are as follows:

- Oral misoprostol (low dose vs high dose*).
- Sublingual misoprostol (low dose vs high dose*).
- Buccal misoprostol (low dose vs high dose*).
- Vaginal misoprostol (low dose vs high dose*).
- Vaginal dinoprostone (regular preparations).
- Controlled-release dinoprostone vaginal pessary.
- Intracervical dinoprostone.
- Laminaria tent (natural osmotic dilator).
- Dilapan-S (synthetic osmotic dilator).
- Single-balloon catheter.
- Double-balloon catheter.
- Balloon catheter plus oral misoprostol.
- Balloon catheter plus vaginal misoprostol.
- Balloon catheter plus oxytocin.
- Nitric oxide donor.
- Extra-amniotic saline infusion.
- Membrane sweeping.
- Mifepristone.
- Placebo.
- Expectant management.
- No treatment.

*Our definition of low dose (≤50 µg every ≥4 hours) aligns with the cut-off used in the Cochrane review by de Vaan *et al*.^26^

**Figure 1:**
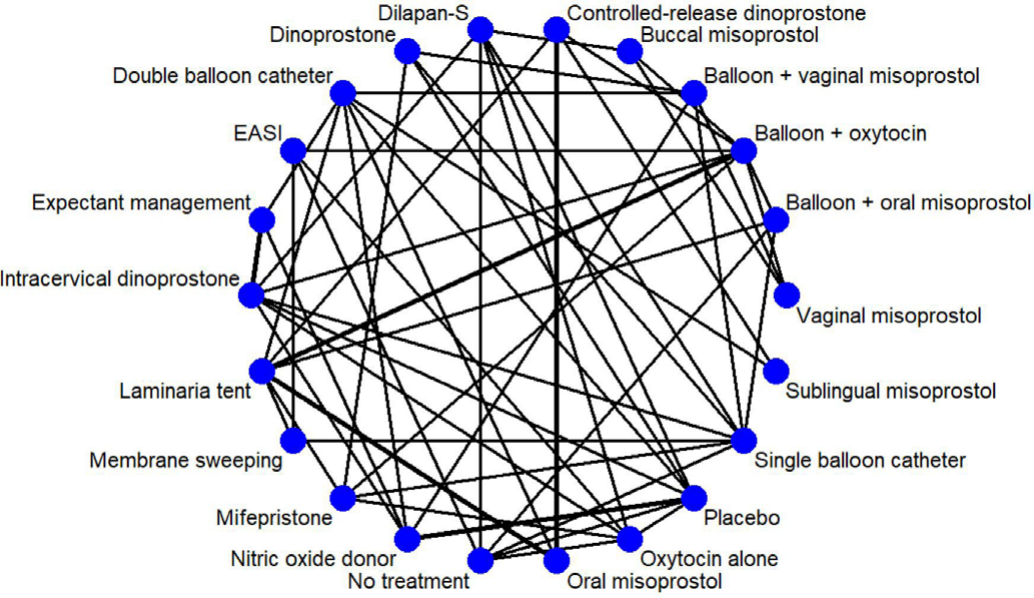
Conceptual network of possible comparisons between induction of labour methods.EASI, extra-amniotic saline infusion.

The final decision to stratify interventions by dosage in the NMA will depend on the number of trials identified for each dose category. This post hoc decision to stratify the data and apply statistical models within each stratum will be made with caution, as excessive stratification of interventions with limited data may result in a sparse network, compromising estimate stability, statistical power and interpretability.^27^ To support this process, a group of non-voting statistical and methodological advisors will be consulted in the selection of intervention nodes, to help ensure methodological feasibility and to prevent inappropriate inclusion or exclusion of interventions. Figure 1 illustrates a conceptual network plot for CIRCLE-NMA.

Studies with two or more arms will be included if at least two arms evaluate interventions of interest, enabling a relevant pairwise comparison.

### Outcome measures

Outcomes for this IPD-NMA will consider those outlined in a core outcome set for RCTs on IOL (COSIOL), which was formulated through an international multi-stakeholder Delphi process.^28^ Outcomes will be selected based on clinical relevance and the general availability of data across existing RCTs. There will be three co-primary outcomes:

1. Vaginal delivery proportion: defined as the proportion of women who achieved vaginal birth, including both spontaneous and instrumental vaginal deliveries. The denominator of the proportion is the number of women contributing data to the analysis.
2. Composite of adverse perinatal outcome: a composite measure of stillbirth, neonatal death, neonatal Apgar score <7 at 5 min, acidosis (pH <7.1), neonatal seizures, hypoxic ischaemic encephalopathy (HIE) of any stage, neonatal intensive care unit (NICU) admission, meconium aspiration syndrome, neonatal infection either clinically suspected (as defined by neonatal antibiotic administration) or proven neonatal infection (culture proven), cord prolapse, endotracheal intubation and/or external cardiac compressions.
3. Composite of adverse maternal outcome: maternal infection (temperature ≥ 38°C at any time during labour or delivery), the use of antibiotics other than group B streptococcus prophylaxis or clinically diagnosed infection (such as endometritis), maternal admission to an intensive care unit, postpartum haemorrhage (≥1000 mL estimated blood loss), uterine rupture, hysterectomy and/or maternal death.

Each composite outcome will be treated as a binary outcome, indicating whether a participant experienced at least one of the component adverse events. Trials will not be required to report all components to contribute to the composite outcomes. If at least one component is reported and an event occurs, the participant will be counted as having experienced the composite outcome. If there is insufficient information to determine whether any component occurred, the composite outcome will be coded as missing. This prevents misclassification bias that could otherwise inflate treatment effects by treating missing outcomes as no events. The extent of missing data across components will be assessed and reported, and sensitivity analyses using data imputation will be conducted where appropriate.

Secondary outcomes will include selected components of the primary composites identified as important by the consumer and public representative focus group.

1. Delivery outcomes:
  - Time from commencement of cervical ripening to delivery.
  - Indication for caesarean delivery, including analysis for fetal compromise or failure to progress (if both fetal compromise and failure to progress apply as indications, fetal compromise prevails).
2. Labour progression outcomes:
  - Uterine hyperstimulation (fetal heart rate abnormalities in the presence of either uterine tachysystole or uterine hypertonus).
  - Additional oxytocin use.
3. Neonatal safety outcomes:
  - Apgar score <7 at 5 min.
  - NICU admission.
4. Maternal safety outcomes:
  - Severe postpartum haemorrhage (≥1000mL estimated blood loss).
  - Maternal admission to the intensive care unit.

Tertiary outcomes will be reported in the supplementary material if there are sufficient data:

1. Delivery outcomes:
  - Cumulative incidence of vaginal birth (time-to-event outcome).
  - Mode of delivery (unassisted vaginal birth or instrumental vaginal birth).
  - Indication for instrumental vaginal birth (if both fetal compromise and other indications such as poor maternal effort or prolonged second stage, medical indications apply as indications, fetal compromise prevails).
  - Use of a second method for cervical ripening after the initially allocated method is unsuccessful.
2. Labour progression outcomes:
  - Uterine tachysystole (more than 5 contractions per 10 min for at least 20 min).^4^
  - Uterine hypertonus (a single contraction lasting >2 min).^4^
  - Meconium-stained amniotic fluid.
3. Neonatal safety outcomes:
  - Apgar score <3 at 5 min.
  - Arterial umbilical cord pH <7.10.
  - Perinatal death (include both stillbirth and neonatal death).
  - HIE of any stage.
  - Neonatal infection, either clinically suspected or proven.
  - Endotracheal intubation.
  - External cardiac compressions.
4. Maternal safety outcomes:
  - Antibiotic administration during labour or delivery.
  - Maternal fever ≥ 38°C during labour or delivery.
  - Uterine rupture.
  - Hysterectomy.
5. Maternal satisfaction, experience and pain*.

*Maternal satisfaction, experience and pain outcomes are subject to data availability and may be prone to selective reporting. As subjective measures, their interpretation and comparability may be limited by differences in assessment tools and the absence of a universally accepted scale. We will explore the extent to which these outcomes can be meaningfully analysed once IPD are obtained.

### Information sources and search strategy

A comprehensive search strategy will be developed in consultation with a librarian. The final strategy will be reviewed and validated using the Peer Review of Electronic Search Strategies checklist to ensure its quality and completeness.^29^ A draft of the search strategy can be found in online supplemental material 1.

Potentially suitable RCTs will be identified from the Cochrane Review on Network Meta-analysis for Induction of Labour.^5^ Databases, including Ovid MEDLINE, Embase via Ovid, Ovid Emcare, Scopus, Cochrane Pregnancy and Childbirth Group’s Trials Register, WHO International Clinical Trials Registry Platform, clinicaltrials.gov (for unpublished, planned and ongoing trial reports) and reference lists of retrieved studies will be searched to identify further trials emerging since the above Cochrane Review was conducted. Two investigators will independently review the identified papers for eligibility, with disagreements to be solved by a third independent reviewer. The principal authors of eligible trials will be invited to contribute their raw data for analysis.

The planned end date for the author enquiry phase is December 2026. The overall Cervical Ripening for Induction of Labour Collaborative Evidence in Network Meta-Analysis (CIRCLE-NMA) study is planned to conclude by December 2027, with a top-up search to be conducted before finalising the manuscript.

### Data collection, management and confidentiality

Eligible RCT investigators will be invited to provide IPD. A fraction of these relevant IPD sets have already been collected for related pairwise IPD meta-analysis projects by our group.^30^,^35^ Regular invitations will be sent to the investigator team of the emerging RCTs asking them to contribute additional data. A draft data dictionary of variables to be sought is provided in online supplemental material 2. De-identified data will be shared either via institutional email using encrypted files or through secure data-sharing platforms. These data will be saved in a password-protected computer and cloud-based platforms at Monash University, Australia. In cases where authors decline to share or the data are unavailable, AD will be extracted from tables in their publications for the AD data NMA component. Wherever possible, data will be extracted based on the intention-to-treat principle.

### Quality and trustworthiness assessment

We will apply the Trustworthiness in RAndomised Clinical Trials (TRACT) checklist to assess the trustworthiness of all eligible studies that do not share IPD.^36 37^ The TRACT checklist is a screening tool that aims to identify and triage RCTs at risk of trustworthiness issues and for which the integrity of the research could, therefore, be poor. The checklist includes seven domains: governance, author group, plausibility of intervention usage, timeframe, drop-out rates, baseline characteristics and outcomes. Two investigators will independently assess RCTs published in full-text form, and disagreements will be resolved by consensus. The TRACT checklist adopts a scoring system for all items, assigning scores as follows: no concerns: 0; some concerns/no information: 1; and major concerns: 2. The total TRACT score for each RCT will be calculated by summing individual item scores. An RCT will be classified as ‘not meeting trustworthiness criteria’ if it meets either of the following conditions: (1) it has a total score of 4 or higher and this score exceeds the median score of RCTs within the relevant IOL comparison group, or (2) it raises serious concerns on any of the designated ‘veto power’ TRACT items, specifically items 3.1, 4.2, 6.2, 6.3 or 7.2. Otherwise, it will be categorised as ‘meeting trustworthiness criteria’. All eligible studies will be categorised into four groups based on trustworthiness assessment and data-sharing status.

For studies that shared IPD, data integrity will be assessed using the ‘IPD Integrity Tool’, which evaluates trustworthiness across eight domains such as data patterns, baseline characteristics and inconsistencies.^38^ Two investigators will independently rate each item as having no, minor or major concerns, providing justifications for their ratings. The tool will be used to help determine whether a trial is included, requires additional information before inclusion, or should be excluded. Any concerns will be discussed with trial authors prior to inclusion in the IPD-NMA. Trials with unresolved major concerns will be excluded from the main analysis.

### Data processing

Data harmonisation: it is likely that baseline characteristics and outcome data have been recorded in a variety of formats and units across trials. To ensure consistency, variables will be recoded, where possible, to align with the same variable names, data categories and measurement units, as defined in our NMA data dictionary.

Data collating: after harmonising the coding of all trial datasets, they will be combined into a single dataset for analysis. Additional variables will be generated within the merged dataset to address specific outcomes, such as the creation of categorical variables.

### Risk of bias and certainty of evidence appraisal

The risk of bias in randomised trials will be evaluated by two investigators using the RoB 2 tool, enhanced with an IPD-informed approach.^39 40^ For the NMA, the overall certainty of evidence will be evaluated by using the Confidence in Network Meta-analysis.^41^ Disagreements will be resolved by consensus.

### PRISMA flowchart

We will create a PRISMA-IPD flow diagram to summarise study selection and data collection, including the number of studies identified, screened, included, excluded (with reasons), and those providing or not providing IPD, along with participant numbers.

### Evidence synthesis and statistical analysis

A SAP will be developed through consensus within the NMA collaboration prior to conducting any analyses. Any limitations in performing specific planned analyses due to insufficient data will be clarified in the final publication. The remainder of this section provides an outline of the analysis approach.

### Type of analysis

In principle, analyses will be categorised based on the trustworthiness classification of the eligible study and data availability.

- IPD meeting our trustworthiness criteria.
- AD meeting our trustworthiness criteria.
- All data meeting our trustworthiness criteria (IPD+AD).
- IPD not meeting our trustworthiness criteria.
- AD not meeting our trustworthiness criteria.
- All data not meeting our trustworthiness criteria (IPD+AD).

Only (A) and (B) will be reported as the primary analysis and will follow the intention-to-treat principle. Analyses (C), (D), (E) and (F) will be presented in the supplementary material. There is the potential for a secondary publication to provide a detailed report on analyses (C) and (F) as part of this project initiative if deemed appropriate.

For the co-primary outcome of ‘vaginal delivery rate’ and non-composite secondary outcomes, treatment effect estimates will be generated for all six analyses (A), (B), (C), (D), (E) and (F) depending on data availability. For the two other co-primary adverse maternal/neonatal outcomes, which are composite outcomes, AD meta-analysis is not possible, and we will only conduct analysis (A).

### Network IPD meta-analyses

We will perform a two-stage contrast-based frequentist IPD-NMA using multivariate random-effects models to compare all available interventions for the primary outcomes, as analysis (A) described above. Effect estimates as ORs and 95% CIs will be presented in a league table for each pairwise comparison. The two-stage approach is selected as the primary analysis strategy because it is more interpretable to clinicians, allows for clearer graphical presentation and offers greater reproducibility. It also enables easier estimation of heterogeneity using familiar metrics and supports the use of restricted maximum likelihood (REML), which is preferred for accurate heterogeneity estimation but not readily available in one-stage models.^42 43^

The transitivity assumption in NMA assumes no systematic differences in effect modifiers across comparisons and is supported here by including only pharmacological and mechanical IOL methods likely to be jointly randomisable in a hypothetical mega-trial.^44^ The distribution of potential effect modifiers across trials and treatment arms will be examined to further probe the assumption and to determine the appropriateness of combining information across trials.^10^

We will further investigate inconsistency, the statistical manifestation of transitivity, in both global and local approaches. We will assess global inconsistency by using a design-by-treatment interaction model^45^ and local inconsistency through the side-splitting approach.^46^ The comparison-adjusted funnel plot will be applied to assess small study effects, based on the assumption that interventions can be ordered by their relative effectiveness in a meaningful way.^47^

The analysis will be executed using the latest stable installation of R, a free software environment for statistical computing and graphics (R Foundation for Statistical Computing, Vienna, Austria).^48^

### Network AD meta-analyses by trustworthiness categorisation

To examine treatment effects across the full scope of available literature on the topic and any comparisons of interest, we will conduct AD network meta-analyses using data extracted from published reports (analyses B, C, D, E, F in ‘Type of Analysis’). For IPD studies, we will also use study-level summary measures (ie, event counts, means and SD). All AD network meta-analyses will be reported in the supplementary material.

### Covariates and subgroups for network meta-analysis

Maternal age and parity will be included as covariates in regression models for the analysis of primary outcomes only. If a study reports only one of these covariates, adjustment will be based on the available covariate for that study.

Potential personalisation factors for subgroup analysis include parity (nulliparous vs multiparous), history of caesarean section, maternal age, body mass index, gestational age at IOL, initial modified Bishop score and indications for IOL. For factors that showed statistically significant interactions between covariates and treatment effects, we will provide subgroup probability rankings. A single heterogeneity parameter will be assumed for each network, and statistical heterogeneity will be assessed using the τ (tau) statistic, and 95% prediction intervals will be reported. Subgroup effects will be investigated for outcomes only if sufficient data are available. Prior to conducting subgroup analyses, we will examine the distribution of potential effect modifiers across treatment comparisons to assess the plausibility of the transitivity assumption.

### Ranking of interventions

**Figure 2:**
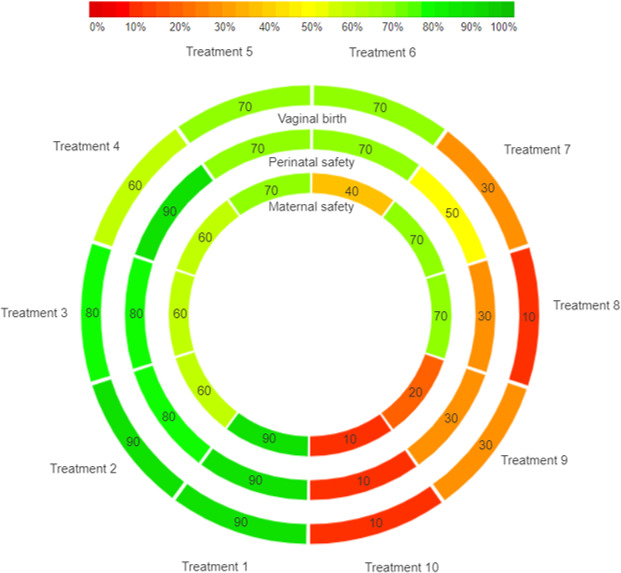
Conceptual rank-heat plot for one subgroup.

The rankings of interventions for each co-primary outcome will be reported separately, based on the treatment effect estimates for each respective outcome using IPD. To determine the treatment hierarchy across effectiveness, perinatal safety and maternal safety for each subgroup, we will calculate the ranking probabilities for each intervention at each rank, using the surface under the cumulative ranking curve (SUCRA) and mean ranks.^49 50^ A rank-heat plot will be created to visualise the rankings, serving as a decision-making tool to communicate the results and assist clinicians in making personalised decisions.^51^ A conceptual figure for demonstration is given in figure 2. This plot includes three circles that stand for the three primary outcomes. The area of each concentric circle is divided by available treatments, and each sector is coloured according to the ranking of the particular treatment at the corresponding outcome. The red colour refers to the smallest SUCRA (0%) which is inferior, values in the middle are yellow, and the green colour symbolises the highest SUCRA (100%) which is superior. This tool can clearly inform the trade-off between effectiveness, perinatal safety and maternal safety, if it exists in any subgroup, to health professionals and women, and a balanced decision regarding which IOL method to use can be made accordingly.

### Planned sensitivity analysis

To evaluate the robustness of results to changes in trial characteristics and choice of analysis methods, sensitivity analyses will be performed for the primary outcome vaginal birth, using IPD classified as meeting our trustworthiness criteria only, provided sufficient data are available once studies have been excluded. These analyses aim to verify the reliability and applicability of the findings, which will include:

- Excluding trials deemed at high risk of bias due to issues with sequence generation, allocation concealment or follow-up.
- Evaluating the possible impact of missing data on the intervention effects using a one-stage approach, where applicable.
- For mixed population studies, we restrict analysis to include data only from those studies that recruited women without a previous caesarean section.
- As-treated analysis.

### Patient and public involvement statement

A focus group of patient and public representatives with lived experience of cervical ripening for IOL has been established to contribute to this project. Patients and the public were first involved during the protocol development stage, providing input on the scope, research objectives and outcomes of interest. Their lived experiences and priorities helped shape the research questions and informed the selection of outcomes that matter most to patients, ensuring the study reflects real-world experiences and concerns. In later stages, the focus group will also be invited to guide the communication and dissemination of findings, including identifying what results should be shared with patient communities, how and in what formats, to ensure accessibility and relevance.

## Ethics and dissemination

### Governance

The IPD-NMA project has been registered with the Human Research Ethics Committee at Monash University (No. 48189). For each included trial, de-identified data will be requested from the original authors, who have obtained local ethics approval to share these data. The chief investigators of the included trials will remain the custodians of their respective trial data. As this study involves analysis of de-identified data from completed trials, there are no direct safety risks to participants. All data will be handled in accordance with relevant ethical and data governance requirements. This project is registered in PROSPERO (CRD420251077464), and the reporting of this study will follow the PRISMA-IPD statement and the PRISMA 2020 statement.^18 19^

### Project management

The NMA collaboration will include representatives from each trial contributing IPD, supported by an advisory group of statistical, methodological and clinical experts, along with consumer representatives. The home team at Monash University will principally oversee data collection, project management, analysis, communication within the collaboration and the drafting of newsletters, reports and publications. All contributing trial investigators will be given opportunities to comment on the initial scope, draft protocol and draft SAP. They will be invited to participate in online meetings as the project progresses. Trial investigators will be approached if there are any questions about their RCTs.

### Dissemination plan

Findings from the CIRCLE-NMA project will be shared through peer-reviewed journal publications, including a protocol paper, main results manuscript and potential secondary analyses. Results will also be presented at relevant national and international conferences. Plain language summaries will be prepared for dissemination to patient groups, public health organisations and professional bodies. Further information about the CIRCLE-NMA project is available at: https://circlenma.com/.^22^ Other potential dissemination strategies include sharing results with healthcare providers (private obstetricians, public hospitals and professional development sessions) via email, social media and direct engagement, and with consumers (eg, general practitioners and midwifery-led clinics) through posters or flyers and social media platforms, as recommended by public and patient representatives of this project.

The opportunity to co-author CIRCLE-NMA papers will be offered based on direct involvement in the project and contribution of trial data. All project advisors and chief investigators providing IPD will be invited to join the ‘CIRCLE-NMA Collaborative’ with the opportunity for group authorship on all relevant project output. The number of authorship opportunities per trial will be based on the sample size of an individual trial and as decided by the review team of this IPD-NMA. Within the ‘CIRCLE-NMA Collaborative’ author group, authors from each contributing trial will be listed in alphabetical order. Results will be published under the home team at Monash University with the ‘CIRCLE-NMA Collaborative’ or by representatives on behalf of the group, as agreed. Draft reports will be circulated to the group for review, input and approval before submission.

As all received IPD remains the property of the original trial groups, the CIRCLE-NMA does not have the authority to share or deposit any data. Individuals who wish to access study data should contact the original trial investigators directly. Key statistical code used for analysis will be curated and deposited in a secure, publicly accessible repository following publication of the primary findings.

## Discussion

The increasing number of trials on cervical ripening in IOL highlights the urgent need for a comprehensive, evidence-based resource that synthesises robust data and provides clear guidance on selecting the most appropriate method based on patient characteristics. However, it is important to recognise that some trials fail to meet trustworthiness standards due to research integrity concerns.^36^ Including such trials without scrutiny risks misleading clinical guidelines. Notably, previous systematic reviews and meta-analyses on similar topics have often overlooked this critical aspect.

By incorporating high-quality IPD and filtering out RCT studies that fail to meet trustworthiness standards, we aim to produce reliable evidence to inform clinical decision-making and identify the most effective interventions. This comprehensive synthesis will play a vital role in recommending optimal cervical ripening methods in IOL and personalising approaches to meet the unique needs of women.

This NMA brings together data from a large number of trials around the world to tackle complex clinical questions that cannot be fully addressed by individual studies or simple pairwise comparisons. Through global collaboration, we aim to establish consensus on the most critical outcomes to redefine practice regarding cervical ripening.

## Supplementary material

10.1136/bmjopen-2025-110091 online supplemental file 1

10.1136/bmjopen-2025-110091 online supplemental file 2
