# Supplementary material for: Methods of cervical ripening in induction of labour: an individual participant data network meta-analysis of randomised controlled trials (CIRCLE-NMA) study protocol
Source: BMJ Open. 2026 Apr 13;16(4):e110091. doi: 10.1136/bmjopen-2025-110091 (PMC13084931; doi:10.1136/bmjopen-2025-110091)
Supplement: online supplemental file 1 [file bmjopen-16-4-s001.docx]

PubMed

(

"cervical ripening"[Mesh] OR "cervical ripening" [tiab]

OR "labor, induced"[mesh]

OR (induction[tiab] AND (labor[tiab] OR labour[tiab] OR cervix[tiab] OR cervical[tiab] OR ripen*[tiab]))

)

AND

(

"misoprostol"[MeSH Terms] OR misoprostol[tiab] OR "prostaglandin E1"[tiab] OR PGE1[tiab]

OR dinoprostone[MeSH Terms] OR dinoprostone[tiab] OR "prostaglandin E2"[tiab]

OR prostin[tiab] OR cervidil[tiab] OR propess[tiab]

OR oxytocin[MeSH Terms] OR oxytocin[tiab]

OR catheter[MeSH Terms] OR catheter[tiab] OR balloon[tiab]

OR "laminaria"[MeSH Terms] OR laminaria[tiab] OR "osmotic dilator"[tiab] OR "dilapan*"[tiab]

OR “nitric oxide”[MeSH Terms] or “nitric oxide”[tiab]

OR EASI[tiab] OR “extra-amniotic saline infusion”[tiab]

OR ("membrane sweep*”[tiab] OR "membrane strip*"[tiab])

OR melatonin[MeSH Terms] OR melatonin[tiab]

OR placebo[tiab]

OR "watchful waiting"[MeSH Terms] OR "expectant management"[tiab]

OR “no treatment”

)

WHO ICTRP

induc* AND labour

induc* AND labor

ClinicalTrials.gov

Expert search

("cervical ripening" OR "labor induced" OR (induction AND (labor OR labour OR cervix OR cervical OR ripenin))) AND (misoprostol OR "prostaglandin E1" OR PGE1 OR dinoprostone OR "prostaglandin E2" OR prostin OR cervidil OR propess OR oxytocin OR catheter OR balloon OR laminaria OR "osmotic dilator" OR dilapan* OR "nitric oxide" OR EASI OR "extra-amniotic saline infusion" OR "membrane sweep*" OR "membrane strip*" OR melatonin OR placebo OR "watchful waiting" OR "expectant management" OR "no treatment") | Interventional studies

Embase & Ovid Emcare

cervical ripening/

labor, induced/
(cervical ripening or labor induced).ti,ab.

(induction and (labor or labour or cervix or cervical or ripen*)).ti,ab.

misoprostol/

oxytocin/

dinoprostone/

catheter/ or exp balloon catheter/

laminaria/

nitric oxide/

melatonin/

watchful waiting/

(misoprostol or "prostaglandin e1" or pge1 or dinoprostone or "prostaglandin e2" or prostin or cervidil or propess or oxytocin or catheter or balloon or laminaria or "osmotic dilator" or dilapan*

or "nitric oxide" or easi or "extra-amniotic saline infusion" or "membrane sweep*" or "membrane* strip" or melatonin or placebo or "expectant management" or "no treatment").ti,ab.

randomized controlled trial.pt. or (randomized or randomised or clinical trial or rct).ti,ab.
