# Supplementary material for: Methods of cervical ripening in induction of labour: an individual participant data network meta-analysis of randomised controlled trials (CIRCLE-NMA) study protocol
Source: BMJ Open. 2026 Apr 13;16(4):e110091. doi: 10.1136/bmjopen-2025-110091 (PMC13084931; doi:10.1136/bmjopen-2025-110091)
Supplement: online supplemental file 2 [file bmjopen-16-4-s002.docx]

# CIRCLE-NMA dATA DICTIONARY

| **CATEGORY** | **VARIABLE** | **CODE** | **TYPE** |
| --- | --- | --- | --- |
| Identification | Study name (last author + year) | study_name |  |
|  | Patient ID in NMA | pid |  |
|  | Patient ID used in original study | oldpid |  |

| Treatment | Pairwise comparison group | comparison | Categorical |
| --- | --- | --- | --- |
|  | Treatment allocation | tr_allocated | Categorical |
|  | Actual treatment received | tr_received | Categorical |

| Baseline Demographics | Age | age | Continuous |
| --- | --- | --- | --- |
|  | BMI | bmi | Continuous |
|  | Gestational age | gestage | Continuous |
|  | Smoking | sm | Categorical  0 = no, 1 = yes |
|  | maternal ethnicity | me | Continuous  1 = African American  2 = Caucasian  3 = Hispanic  4 = Other  5 = Asian |
|  | Parity | parity | Continuous |
|  | Parity code | parcode | Categorical  0 = nulliparous  1 = multiparous |
|  | Indication for IOL | iol_ind | Categorical (text)   - other/unknown - hypertensive disorders - post-term labour - diabetes mellitus - oligohydramnios - IUGR - premature rupture of membranes (PROM) - decreased fetal movements - elective/maternal request - >1 indication   APH |
|  | Initial Bishop score | bs | Continuous |
|  | Medications given during labour | meds | Categorical   - no medications - antihypertensives - aspirin - heparin - antihypertensives + aspirin - antihypertensives + heparin - aspirin + heparin - terbutaline - magnesium sulphate - magnesium sulphate + antihypertensives |
|  | medications given during labour | meds_binary | Categorical  0 = no, 1 = yes |
|  | history of caesarean section | histcs | Categorical   - 0 = no, 1 = yes |

| Primary Outcomes | Mode of delivery | mod | Categorical  0 = caesarean section  1 = vaginal instrumental  2 = vaginal unassisted |
| --- | --- | --- | --- |
|  | Spontaneous vaginal birth | svb | Categorical  0 = no, 1 = yes |

| Secondary perinatal Outcomes | fetal death | fdeath | Categorical  0 = no, 1 = yes |
| --- | --- | --- | --- |

| Secondary  Delivery  Outcomes | indication for vacuum or forceps extraction | vefeind | Categorical (text)   - not applicable - failure to progress in second stage - fetal distress - fetal distress and failure to progress in second stage - maternal complications/other - not included |
| --- | --- | --- | --- |
|  | indication for vacuum or forceps extraction due to fetal distress | vefeind_distress | Categorical  0 = no, 1 = yes |
|  | indication for caesarean section | csind | Categorical (text)   - not applicable - failure to progress - fetal distress - fetal distress and failure to progress - maternal complications/other - failed induction - cephalo-pelvic disproportion - cord prolapse - placental abruption |
|  | Indication for caesarean section due to fetal distress | csind_distress | Categorical  0 = no, 1 = yes |
|  | induction to birth interval in hours | ind_birth | Continuous |

| Secondary Labour Progression Outcomes | oral misoprostol dose in mcg | omdose | Continuous |
| --- | --- | --- | --- |
|  | number of doses of oral misoprostol given | omdoseno | Continuous |
|  | vaginal dinoprostone dose in mg | vddose | Continuous |
|  | number of doses of vaginal dinoprostone given | vddoseno | Continuous |
|  | change in induction method from allocated | iol_change | Categorical  0 = no, 1 = yes |
|  | Failure of cervical ripening device used | fail | Categorical  0 = no, 1 = yes |
|  | use of oxytocin during labour | oxy | Categorical  0 = no, 1 = yes |
|  | initial modified Bishop score | mbs_initial | Continuous |
|  | maximum modified Bishop score recorded | mbs_max | Continuous |
|  | change in modified Bishop score from initial to maximum | mbs_change | Continuous |
|  | uterine hyperstimulation | uh | Categorical  0 = no, 1 = yes |
|  | Uterine tachysystole | ts | Categorical  0 = no, 1 = yes |
|  | Meconium liquor | mec | Categorical  0 = no, 1 = yes |

| Secondary maternal safety outcomes | maternal temperature ≥38C | temp | Categorical  0 = no, 1 = yes |
| --- | --- | --- | --- |
|  | Stage maternal antibiotics given at | abx_stage | Categorical  0 = no  1 = before onset of labour  2 = during labour and delivery  3 = after delivery (UTI or wound infection) |
|  | maternal antibiotics given | abx | Categorical  0 = no, 1 = yes |
|  | maternal infections | inf_maternal | Categorical (text)   - none - endometritis - wound infection - intraamniotic infection - urinary tract - other (eg. pneumonia) |
|  | maternal infections | inf_maternal_binary | Categorical  0 = no, 1 = yes |
|  | maternal analgesia use during labour | analg | Categorical (text)  0 = none  1 = epidural  2 = spinal  3 = local anaesthetic  4 = other  5 = epidural and opioids  6 = opioids  7 = nitrous oxide  8 = >1 |
|  | maternal analgesia use during labour | analg_binary | Categorical  0 = no, 1 = yes |
|  | maternal side effects | mse | Categorical  0 = none  1 = vomiting  2 = nausea  3 = diarrhoea  4 = shivering  5 = pyrexia  6 = other  7 = nausea and vomiting and shivering  8 = nausea and vomiting  9 = vomiting and shivering  10 = nausea and diarrhoea  11 = nausea and shivering |
|  | maternal side effects | mse_binary | Categorical  0 = no, 1 = yes |
|  | Maternal death | mdeath | Categorical  0 = no, 1 = yes |
|  | Maternal icu admission | micu | Categorical  0 = no, 1 = yes |
|  | Estimated blood loss | ebl | Continuous |
|  | post-partum haemorrhage (>500mL) | pph500 | Categorical  0 = no, 1 = yes |
|  | post-partum haemorrhage (>1000mL) | pph1000 | Categorical  0 = no, 1 = yes |
|  | Urinary tract infection | uti | Categorical  0 = no, 1 = yes |
|  | Bowel obstruction | bo | Categorical  0 = no, 1 = yes |
|  | Thromboembolic event | throm | Categorical  0 = no, 1 = yes |
|  | Hypertensive disorder | htnd | Categorical  0 = no, 1 = yes |
|  | Pre-eclampsia/HELLP | preecl | Categorical  0 = no, 1 = yes |
|  | Other postpartum condition requiring treatment | othercx | Categorical  0 = no, 1 = yes |
|  | Uterine rupture | ur | Categorical  0 = no, 1 = yes |

| Secondary Neonatal Morbidity Outcomes | APGAR score recorded as <7 at 5 minutes | apgar | Categorical  0 = no (i.e. APGAR 7-10 at 5 minutes)  1 = yes (i.e. APGAR 1-6 at 5 minutes) |
| --- | --- | --- | --- |
|  | Premature rupture of membranes | prom | Categorical  0 = no, 1 = yes |
|  | Admission to neonatal intensive care unit | nicu | Categorical  0 = no, 1 = yes |
|  | Neonatal seizure | ns | Categorical  0 = no, 1 = yes |
|  | Severe neonatal respiratory compromise | nresp | Categorical  0 = no, 1 = yes |
|  | Neonatal infection | inf_neonatal | Categorical  0 = no, 1 = yes |
|  | Neonatal death/stillbirth | stillbirth | Categorical  0 = no, 1 = yes |
|  | Arterial umbilical cord pH <7.10) | artph | Categorical  0 = no (i.e. pH 7.10 or greater)  1 = yes (i.e. pH <7.10) |
|  | Number of foetuses | nf | Continuous |
|  | Multiple foetuses | mf | Categorical  0 = no, 1 = yes |
|  | Meconium stained liquor | msl | Categorical  0 = no, 1 = yes |
|  | Maternal retained placenta | rp | Categorical  0 = no, 1 = yes |
