# Supplementary material for: Methods of cervical ripening in induction of labour: an individual participant data network meta-analysis of randomised controlled trials (CIRCLE-NMA) study protocol
Source: BMJ Open. 2026 Apr 13;16(4):e110091. doi: 10.1136/bmjopen-2025-110091 (PMC13084931; doi:10.1136/bmjopen-2025-110091)
Supplement: Supplementary file 3 [file bmjopen-2025-110091.reviewer_comments.pdf]

PEER REVIEW HISTORY

BMJ Open publishes all reviews undertaken for accepted manuscripts. Reviewers are asked to complete a checklist review form and are provided with free text boxes to elaborate on their assessment. These free text comments are reproduced below.

ARTICLE DETAILS

Title (Provisional)

Methods of Cervical Ripening in Induction of Labour: An Individual Participant Data Network Meta-Analysis of Randomised Controlled Trials (CIRCLE-NMA) - Study Protocol

Authors

Au, Nicole; Patabendige, Malitha; Libesman, Sol; Wang, Rui; Gurrin, Lyle; Mol, Ben W; Li, Wentao; CIRCLE-NMA Collaborative, CIRCLE-NMA Collaborative; Jones, Madeleine; Chan, Fei; Tan, Sheareen; Zhang, Jun; Grobman, William A; Mundle, Shuchita; Haas, David; Allotey, John; Galadanci, Hadiza; Pacagnella, Rodolfo c; Seidler, Anna Lene; Karahalios, Amalia; Hunter, Kylie Elizabeth; Sheehan, Penelope M; Berghella, Vincenzo; Hosier, Hillary; Romero, Marino; Aggarwal, Neelam; Pasrija, Shikha; Henry, Amanda; Jozwiak, M; Subramaniam, Akila; Tita, Alan; Baidya, Jahar Lal; Policiano, Catarina; Goonewardene, Malik; Bagga, Rashmi; Sass, Nelson; Haugland, Birte; Osoti, Alfred O; Wallström, Tove; Al-Ibraheemi, Zainab; Anand, Amandeep; Deshmukh, Varsha L; Daellenbach, Patrick; Kruit, Heidi; Young, David; Cheng, Shi-Yann; Cecatti, Jose Guilherme; Waterstone, Mark; Manjunath, Attibele Palaksha; Pennell, Craig; Saad, Antonio; Rozenberg, P.; Edwards, Rodney K.; Ng, Yang Huang Grace; le Roux, Paul; Marions, Lena; Van Gemund, Nicollette; Beckmann, Michael; Dodd, Jodie M; Mathews, Jiji E; Løkkegaard, Ellen Christine Leth; Wise, MR; Diguisto, Caroline; Vayssière, C; ten Eikelder, MLG; Liu, Xiaohua; Le Gouge, Amélie; Thompson, John MD; Perrotin, Franck; Stannard, Amelia; Lelliott, Jamielee; Kongvongsa-Patel, Laura

---

VERSION 1 - REVIEW

**Reviewer**	**1**
**Name**	**Williams, Linda**
**Affiliation**	**University of Edinburgh, Usher Institute**
**Date**	**19-Dec-2025**
**COI**	**None**

Excellent protocol, well written and comprehensive. A few minor points.

Second & third co-primary outcomes will need to be treated very carefully if not all parts of the composite measure are required from trials.

If a second method is used following the failure of the first, which method is being considered in the analysis? Just using ITT? Any consideration of a sensitivity analysis based on final IOL method?

Sample size, no mention of a minimum sample size for the IPD. Is this because you already have sufficient from your previous work? Similarly, do you have a minimum IPD and number of datasets to explore subgroups and clinical coefficients?

VERSION 1 - AUTHOR RESPONSE

A. Reviewer comment 1	Second & third co-primary outcomes will need to be treated very carefully if not all parts of the composite measure are required from trials.
B. Response	Thank you for the comment. We understand the concern that composite outcomes may be biased if not all components are available from each trial. First of all, we take a conservative approach: "at least one component is reported and an event occurs, the participant will be counted as having experienced the composite outcome." This is already stated in the original protocol. And to improve clarity, we have clarified in the protocol how we will handle records/trials where not all components of the composite outcome are reported. Where there is insufficient information to determine whether any component occurred, the composite will be coded as missing (i.e. we will not assume "no event" when no components are reported). This avoids misclassification bias that could potentially exaggerate treatment effects by incorrectly classifying missing data as no events. However, based on our experience conducting pairwise IPD meta-analyses, it is very rare for a trial to report none of the neonatal or maternal safety components.<br>Second, most individual components in the composite outcomes are correlated, for example, conditions like neonatal seizures and HIE are very likely to lead to NICU admission. In cases where seizures or HIE were missing, they will still be captured in NICU admission, and further into the composite neonatal outcome as a positive event.<br>Also, we have used this composite outcome design in several published pairwise IPD meta-analyses, and sensitivity analyses supported the robustness

	of this method. Therefore, we are confident that the current neonatal and maternal composite outcomes will provide an adequate measure.[1,2,3,4,5,6]<br>We will quantify and report the extent and pattern of missingness across components by trial, and perform pre-specified sensitivity analyses to assess robustness in this IPD network meta-analysis.<br>In addition, several components will be analysed individually as secondary outcomes to assess treatment effects for each outcome separately.
C. Changes made	"If there is insufficient information to determine whether any component occurred, the composite outcome will be coded as missing for the participant/trial, this prevents misclassification bias that could otherwise inflate treatment effects by treating missing outcomes as no events."
D. Location of changes	P.11 Methods

A. Reviewer comment 2	If a second method is used following the failure of the first, which method is being considered in the analysis? Just using ITT? Any consideration of a sensitivity analysis based on final IOL method?
B. Response	Thank you for the comment. In our primary analysis, we will follow the intention-to-treat (ITT) principle. We will also report descriptive statistics on treatment changes using the variables "failure of cervical ripening device used" and "change in induction method from allocated". In addition, we have pre-specified an as-treated analysis as a planned sensitivity analysis (see p.17 of the original protocol under "Planned sensitivity analyses"), in which participants will be analysed according to the actual cervical ripening method received.
C. Changes made	No changes made.
D. Location of changes	No changes made.

A. Reviewer comment 3	Sample size, no mention of a minimum sample size for the IPD. Is this because you already have sufficient from your previous work? Similarly, do you have a minimum IPD and number of datasets to explore subgroups and clinical coefficients?

B. Response	Thank you for raising this. We did not specify a minimum IPD sample size because this is largely dependent on data sharing, rather than our ability to recruit to a target. While we already have substantial IPD for several key interventions from our previous work, the amount of IPD available for newly added interventions will depend on how many eligible trials ultimately share data. We will include all IPD available.<br>Treatment-covariate interaction and subgroup analyses will be conducted only where sufficient IPD are available. The decision to undertake these analyses will be based on examining the distribution of potential effect modifiers across treatment comparisons to assess the plausibility of the transitivity assumption, rather than on the number of IPD received. We have also planned sensitivity analyses to assess the potential impact of missing data on intervention effects, using a one-stage approach where applicable. These have already been specified in the protocol, therefore, no changes will be made in response to this comment.<br>Importantly, we will use the GRADE approach to rate the certainty of evidence in interpreting results, where precision of the estimate is an important component.
C. Changes made	No changes made.
D. Location of changes	No changes made.

- End -

References

1. Jones MN, Palmer KR, Pathirana MM, Cecatti JG, Filho OBM, Marions L, et al. Balloon catheters versus vaginal prostaglandins for labour induction (CPI Collaborative): an individual participant data meta-analysis of randomised controlled trials. Lancet. 2022;400(10364):1681-92.
2. Kemper JI, Li W, Goni S, Flanagan M, Weeks A, Alfirevic Z, et al. Foley catheter vs oral misoprostol for induction of labor: individual participant data meta-analysis. Ultrasound Obstet Gynecol. 2021;57(2):215-23.
3. Peel MD, Croll DMR, Kessler J, Haugland B, Pennell CE, Dickinson JE, et al. Double- vs single-balloon catheter for induction of labor: Systematic review and individual participant data meta-analysis. Acta Obstet Gynecol Scand. 2023;102(11):1440-9.
4. Patabendige M, Chan F, Vayssiere C, Ehlinger V, Van Gemund N, le Cessie S, et al. Vaginal misoprostol versus vaginal dinoprostone for cervical ripening and induction of labour: An individual participant data meta-analysis of randomised controlled trials. Bjog. 2024;131(9):1167-80.

5. Chan F, Patabendige M, Wise MR, Thompson JMD, Sadler L, Beckmann M, et al. Inpatient vaginal dinoprostone vs outpatient balloon catheters for cervical ripening in induction of labor: An individual participant data meta-analysis of randomized controlled trials. Acta Obstet Gynecol Scand. 2025;104(6):1041-55.
6. Patabendige M, Chan F, Wise MR, Thompson JMD, Beckmann M, Saad AF, et al. Outpatient or Inpatient Setting for Cervical Ripening Before Induction of Labour: An Individual Participant Data Meta-Analysis. Bjog. 2025.
